# The Antileukemic and Anti-Prostatic Effect of Aeroplysinin-1 Is Mediated through ROS-Induced Apoptosis via NOX Activation and Inhibition of HIF-1a Activity

**DOI:** 10.3390/life12050687

**Published:** 2022-05-05

**Authors:** Shou-Ping Shih, Mei-Chin Lu, Mohamed El-Shazly, Yu-Hsuan Lin, Chun-Lin Chen, Steve Sheng-Fa Yu, Yi-Chang Liu

**Affiliations:** 1 Doctoral Degree Program in Marine Biotechnology, National Sun Yat-Sen University, Kaohsiung 804, Taiwan; d065620003@nsysu.edu.tw; 2 Doctoral Degree Program in Marine Biotechnology, Academia Sinica, Taipei 115, Taiwan; 3 Graduate Institute of Marine Biology, National Dong Hwa University, Pingtung 944, Taiwan; jinx6609@nmmba.gov.tw (M.-C.L.); 810863001@gms.ndhu.edu.tw (Y.-H.L.); 4 National Museum of Marine Biology & Aquarium, Pingtung 944, Taiwan; 5 Department of Pharmacognosy, Faculty of Pharmacy, Ain-Shams University, Organization of African Unity Street, Cairo 11566, Egypt; mohamed.elshazly@pharma.asu.edu.eg; 6 Department of Pharmaceutical Biology, Faculty of Pharmacy and Biotechnology, German University in Cairo, Cairo 11835, Egypt; 7 Department of Biological Sciences, National Sun Yat-Sen University, Kaohsiung 804, Taiwan; 8 Department of Biotechnology, Kaohsiung Medical University, Kaohsiung 807, Taiwan; 9 Graduate Institute of Natural Products, College of Pharmacy, Kaohsiung Medical University, Kaohsiung 807, Taiwan; 10 Institute of Chemistry, Academia Sinica, Taipei 115, Taiwan; 11 Division of Hematology-Oncology, Department of Internal Medicine, Kaohsiung Medical University Hospital, Kaohsiung 807, Taiwan; 12 Department of Internal Medicine, Faculty of Medicine, College of Medicine, Kaohsiung Medical University, Kaohsiung 807, Taiwan; 13 Cellular Therapy and Research Center, Kaohsiung Medical University Hospital, Kaohsiung 807, Taiwan

**Keywords:** apoptosis, topoisomerase, reactive oxygen species (ROS), heat shock protein 90 (HSP90), aeroplysinin-1 (Apl-1), NADPH oxidases (NOXs)

## Abstract

Aeroplysinin-1 is a brominated isoxazoline alkaloid that has exhibited a potent antitumor cell effect in previous reports. We evaluated the cytotoxicity of aeroplysinin-1 against leukemia and prostate cancer cells in vitro. This marine alkaloid inhibited the cell proliferation of leukemia Molt-4, K562 cells, and prostate cancer cells Du145 and PC-3 with IC_50_ values of 0.12 ± 0.002, 0.54 ± 0.085, 0.58 ± 0.109 and 0.33 ± 0.042 µM, respectively, as shown by the MTT assay. Furthermore, in the non-malignant cells, CCD966SK and NR8383, its IC_50_ values were 1.54 ± 0.138 and 6.77 ± 0.190 μM, respectively. In a cell-free system, the thermal shift assay and Western blot assay verified the binding affinity of aeroplysinin-1 to Hsp90 and Topo IIα, which inhibited their activity. Flow cytometry analysis showed that the cytotoxic effect of aeroplysinin-1 is mediated through mitochondria-dependent apoptosis induced by reactive oxygen species (ROS). ROS interrupted the cellular oxidative balance by activating NOX and inhibiting HIF-1α and HO-1 expression. Pretreatment with *N*-acetylcysteine (NAC) reduced Apl-1-induced mitochondria-dependent apoptosis and preserved the expression of NOX, HO-1, and HIF-1a. Our findings indicated that aeroplysinin-1 targeted leukemia and prostate cancer cells through multiple pathways, suggesting its potential application as an anti-leukemia and prostate cancer drug lead.

## 1. Introduction

According to the global cancer statistics in 2020, prostate cancer ranks as the second most common cancer in males in countries with a higher Human Development Index (HDI), while it ranks first in countries with a lower HDI [1]. Prostate cancer is the fifth leading cause of cancer death in men worldwide [2]. Androgen deprivation therapy (ADT) effectively inhibits the growth of prostate cancer tumors, but it can lead to metastatic castration-resistant prostate cancer (mCRPC) in the process of 18 to 24 months of treatment, resulting in a significant increase in patient mortality [3,4]. According to previous clinical evidence, the abnormal activation of epithelial–mesenchymal transition (EMT) is associated with tumor resistance and aggressiveness, and plays an important role in mCRPC [5]. Currently, the most common treatment is a combination therapy of docetaxel and cabazitaxel, but most patients develop drug resistance [6,7]. Although recent years have witnessed some breakthroughs and advancements in the treatment strategies, there is still no effective treatment against metastatic prostate cancer. Therefore, the prevention of cancer spread and tumor metastasis is still an important challenge in clinical research, and there is no approved drug for EMT. There is an urgent need to develop drugs that can effectively inhibit EMT and metastatic prostate cancer.

Another deadly cancer that affects millions of patients worldwide is leukemia. The number of new adult leukemia cases in the United States is approximately 13.8 per 100,000 per year [8]. Currently, chemotherapy is still the treatment of choice for acute lymphoblastic leukemia (ALL), despite the use of allogeneic hematopoietic stem cell transplantation. The prognosis of adult ALL patients is usually poor, and the overall survival rate is less than 45% [9]. Although surface antigen-targeted therapy can effectively treat B-cell ALL (B-ALL) patients, there is a high recurrence rate for T-cell ALL (T-ALL) patients. However, when combined with other chemotherapeutic agents, this therapy is useful for treating recurrent T-ALL patients [10,11]. Therefore, understanding the mechanism of leukemia and chemoresistance is one of the most important steps in the development of drugs for the treatment of T-ALL patients. The latest evidence shows that the PI3K/Akt/mTOR and Jak/STAT pathways play an important role in inhibiting the apoptosis of hematopoietic cancer cells [12,13,14]. The PI3K/Akt/mTOR signaling pathway is overactivated in 92% of T-ALL cell lines, and is also dysregulated in 42% of primary prostate cancer samples and 100% of CRPCs [15,16]. In prostate cancer and leukemia, many signaling pathways are regulated by active AKT, including extracellular signal-regulated kinase 1/2 (ERK1/2), signal transducers and activators of transcription 3 (STAT3), forkhead box O (FOXO) transcription factors, and Notch1 pathway [15,16]. These cascades can promote the proliferation, progression, and survival of tumor cells, and also play an important role in the invasion, metastasis, and drug resistance of cancer cells.

Heat shock protein 90 (Hsp90) is one of the most critical ATP-dependent chaperone proteins that regulate the folding and maturation of more than 300 client proteins, and also regulates cancer-critical mechanisms, such as cancer cell growth, proliferation, angiogenesis, metastasis, and apoptosis, and drug resistance [17]. HSP90 is involved in the stabilization of transforming growth factor β (TGF-β), mitogen-activated protein kinases (MAPKs), AKT/PI3K, and WNT pathways [18]. HSP is highly expressed in leukemia patients and prostate cancer, and is involved in the relapse or death of patients with resistance to chemotherapy and radiotherapy [19,20]. While there are currently no FDA-approved drugs targeting HSP90, the current pre-clinical and clinical studies suggest that HSP90 inhibitors are promising combinatorial partners for receptor tyrosine kinase (RTK) blockade [18]. It was shown to possess antiproliferative effects in many cancers, including breast, prostate, lung, pancreatic, gastric, colorectal, and leukemia [17,21]. In addition, several studies showed that HSP90 inhibitors interacting with topo IIα can overcome cancer drug resistance and reduce side effects, as well as produce synergistic antiproliferative effects [22].

The role of reactive oxygen species (ROS) is to regulate various cell functions, such as cellular migration and proliferation. Many studies showed that ROS are related to cancer development, but the exact mechanism by which ROS regulate cancer is still not fully understood. ROS and oxidative stress are related to the occurrence and progression of cancer by inducing DNA mutation or DNA damage, genome instability, and the tendency of cell proliferation [23]. However, excessive ROS levels can destroy cellular components, including proteins, lipid bilayers, and chromosomes, leading to cell death. ROS play an important role in a wide range of biological processes, including signal transduction, enzymes regulation, protein channels, and transcription factor activities, such as epidermal growth factor receptor (EGFR), PI3K/AKT, Ras/MAPK pathway [24]. The two main sources of ROS are the mitochondrial respiratory chain, which produces ROS as by-products, and the active nicotinamide adenine dinucleotide phosphate oxidase (NOX), whose main function is to produce ROS [25]. NOX1-3 activation mainly produces O_2_^−^, and NOX4 produces a large amount of H_2_O_2_ [26]. Previous reports have shown that in comparison with normal cells, the increase in ROS production in prostate cancer, lymphoma, and myeloid leukemia cells is mainly caused by NOX enzymes, and their functions are essential for the malignant phenotype of prostate cancer cells [27,28]. The activation of the NOX enzymes requires the translocation of cytoplasmic regulatory proteins to the plasma membrane to form a complex with flavocytochrome b558, which transfers electrons to oxygen molecules through NADPH to produce superoxide radicals [29,30].

**Figure 1:**
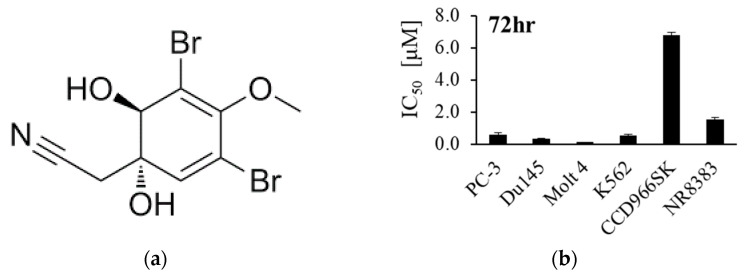

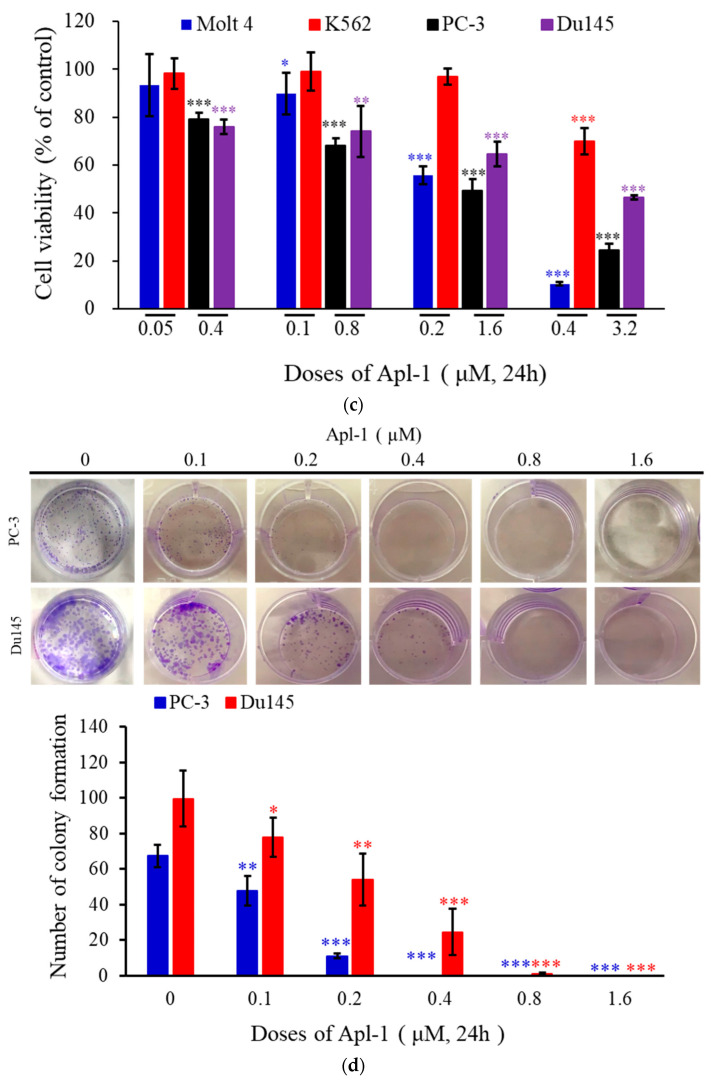
Aeroplysinin-1 inhibited leukemia and prostate cancer cells proliferation. (**a**) Chemical structure of aeroplysinin-1. (**b**) IC_50_ of aeroplysinin-1 against four cancer cell lines and two nonmalignant cells. (**c**) MTT assay was used to measure the cell viability following treatment with aeroplysinin-1 at the indicated concentrations for 24 h. (**d**) PC-3 and Du145 cells were incubated with the indicated concentrations of aeroplysinin-1 for 14 days, and then PC-3 and Du145 cell colonies were counted. The results are shown as mean ± SD of three independent experiments. * *p* < 0.05, ** *p* < 0.01, *** *p* < 0.001, control vs. Apl-1 group.

In previous studies, marine bromine-containing derivatives were found to possess numerous biological potentials, including the 2-(5-bromo-1H-indol-3-yl)-*N*,*N*-dimethylethanamine, and 2-(5-bromo-1H-indol-3-yl)-*N*,*N*-dimethyl-2-oxoacetamide showed significant antidepressant-like effects [31]. Pseudoceramines A–D and ceratinadins A-E demonstrated antimalarial and antibacterial activity [32,33]. Spermatinamine and aplysamine 6 inhibited isoprenylcysteine carboxyl methyltransferase (Icmt ) as an anticancer target, and fistularin-3 derivatives regulated DNA methyltransferase 1 (DNMT1) activity [34,35]. Aerothionin and subereaphenol C showed effective cytotoxic activity against human cervical cancer cells HeLa, and aeroplysinin-2 showed potent anti-migratory activity against the human breast cancer cell line MDA-MB-231 [36]. Aeroplysinin-1 (Apl-1) is a brominated isoxazoline alkaloid isolated from the marine sponge *Pseudoceratina* species (Figure 1a) [37,38]. Previous reports have indicated that this compound plays an important role in the chemical defense mechanism of sponges. In the biological assay, it showed antibacterial, antiviral, antiangiogenesis, antitumor, and anti-inflammatory effects [39]. Apl-1 inhibited cell growth and exhibited cytotoxic effects on a variety of tumor cells (cervical cancer, fibrosarcoma, colorectal cancer, and leukemia cancer), through the suppression and regulation of cell growth and survival-related proteins Akt and ERK [39]. It also induced γ-H_2_AX (a marker protein of DNA damage), activated caspase 3, and cleaved PARP (a marker protein of apoptosis) and p21 (cell cycle-related protein) [39,40,41]. Apl-1 inhibited the protein tyrosine kinase activity of the EGFR kinase complex of human breast cancer cells [41]. Apl-1 analogs inhibited EGFR tyrosine kinase activity, while Apl-1 did not show any significant inhibitory effect [42]. Nevertheless, Apl-1 potential as a cytotoxic agent is not extensively evaluated. Therefore, the purpose of this study is to evaluate the cytotoxic effect and anti-metastatic mechanism of action of Apl-1 on leukemia and prostate cancer cells. The effect of Apl-1 on the regulation of Nox/ROS was also examined.

## 2. Materials and Methods

### 2.1. Chemicals and Biological Materials

The cell lines were purchased from the American Type Culture Collection (ATCC, Manassas, VA, USA). RPMI 1640 was the growing medium for Molt4 and K562. The DU145 and CCD-966SK cells were cultured in the minimum essential medium. Ham’s F-12 medium was the growing medium for PC-3 and NR8383 cells. All media were supplemented with glutamine (2 mM), antibiotics (100 μg/mL of streptomycin and 100 units/mL of penicillin), and 10% fetal bovine serum. The cell lines were kept in a 5% CO_2_ humidified atmosphere at 37 °C. GibcoBRL (Gaithersburg, MD, USA) was the supplier for trypan blue, streptomycin, RPMI 1640 medium, fetal calf serum (FCS), and penicillin. G. Sigma-Aldrich (St. Louis, MO, USA) was the supplier of dimethyl sulfoxide (DMSO) and all other chemicals. Molecular Probes and Invitrogen technologies (Carlsbad, CA, USA) were the sources of rhodamine 123 dye and the dihydroethidium (DHE) of fluorescein. Antibodies against Slug, vimentin, p-Akt (Ser473), EGFR, p-mTOR (Ser2448), XIAP, Bcl-2, cleavage PARP, PI3K, NOX2, NOX4, catalase, HO-1, and Topo II α antibody were purchased from Santa Cruz Biotechnology (Santa Cruz, CA, USA). *N*-Cadherin antibody was purchased from ABclonal Technology (Taipei, Taiwan). Sigma-Aldrich (St. Louis, MO, USA) was the source of antibodies against MMP2 and MMP9. Antibodies against cleaved caspases-3 were obtained from Cell Signaling Technologies (Beverly, MA, USA). HIF1 (Halpha111a), MnSOD, Cu/ZnSOD, HSP90, and HSP70 antibodies were purchased from Enzo life sciences (Taipei, Taiwan).

### 2.2. Cell Proliferation Activity Assay

MTT assay was used for the cell proliferation assay. Cancer cells were plated in a 96-well plate (7 × 104 cells for each well) and were cultured in different media [43,44]. At the 24, 48, or 72 h after seeding, 50 µL of MTT (thiazole blue tetrazolium bromide, Sigma-M2128) was added for staining. The absorbance values at 570 and 620 nm were measured via the ELISA reader (Anthoslabtec instrument, Salzburg, Austria) after a 2–4 h incubation at 37 °C. The IC_50_ value (the concentration that causes 50% inhibition) was calculated.

### 2.3. The Stock Solution of Apl-1

Apl-1 was obtained from the Graduate Institute of Marine Biotechnology, National Dong Hwa University. The Apl-1 was prepared by first dissolving with DMSO at a concentration of 10 mM, and then diluting the cell medium to different concentrations (0.05 to 10 μM).

### 2.4. Annexin V/PI Apoptosis Assay

The externalization of phosphatidylserine (PS) and membrane integrity was determined using the annexin V-FITC staining kit [38]. Strong Biotech Corporation (Taipei, Taiwan) was the supplier of annexin V-FITC/PI (propidium iodide) stain. In brief, 5 × 10^5^ cells were grown in 35 mm diameter plates and were treated with different concentrations of compounds or 0.1% DMSO for 24 h. To label the cells, annexin V-FITC (10 μg/mL), and PI (20 μg/mL) were used. After labeling, all plates were washed with a binding buffer and were harvested. The cells were resuspended in the binding buffer (2 × 105 cells/mL) before analysis by FACS-Caliburflow cytometer (Beckman Coulter, Taipei, Taiwan) and WinMDI software. Approximately 10,000 cells were counted for each determination.

### 2.5. Determination of ROS Generation and MMP Destruction

MMP disruption and ROS generation were examined by fluorescent rhodamine 123 cationic dye (5 μg/mL) and dihydroethidium (DHE, 5 mM), respectively. After treating the cells with different concentrations of Apl-1, the cells were labeled with specific fluorescent dyes for 30 min. PBS was used for washing. The changes in MMP and ROS were determined using flow cytometry.

### 2.6. Fluorescence Microscopic Analysis

The drug-treated cells (Molt4 and K562) were placed on glass slides by cytospinning at 800 rpm for 5 min. PC3 and Du145 cells were cultured on the glass slide at 37 °C in 5% CO2 for 24 h and then treated with drugs. The cells were fixed with 4% paraformaldehyde in PBS buffer (pH 7.3) for 30 min at 4 °C. The cells were infiltrated in ice methanol for 3 min and were washed with PBS. DAPI (1 μg/mL) was used as a counterstain to stain the nucleus. The cells were examined with an FV1000 confocal laser scanning microscope (Olympus, Tokyo, Japan).

### 2.7. Western Blot Analysis

Cells were washed twice with PBS and were treated with RIPA lysis buffer (1% Nonidet P-40, 1× PBS, 0.1% sodium dodecyl sulfate (SDS), 0.5% sodium deoxycholate, 1 mM sodium orthovanadate, 100 μg/mL phenylmethylsulfonyl fluoride, and 30 μg/mL aprotinin) (all chemicals were obtained from Sigma-Aldrich) for 30 min to obtain the cell lysates [45]. The lysates were collected and centrifuged at 13,000× *g* for 30 min at 4 °C. The protein concentration in the supernatant was determined using the BCA protein assay kit (Pierce, Rockford, IL, USA). The same amount of protein was passed through SDS-polyacrylamide gel electrophoresis (10%, or 12%), which were then electrotransferred to a PVDF membrane. TBST buffer containing 5% skimmed milk powder solution (20 mM Tris-HCl, pH 7.4, 150 mM NaCl, and 0.1% Tween 20) was used to block the membrane for 1 h, and the membrane was then washed with TBST buffer. The protein expressions were monitored using specific antibodies. The proteins were detected by an enhanced chemiluminescence kit (Pierce, Rockford, IL, USA).

### 2.8. Protein Thermal Shift Detection

Protein thermal shift was detected according to the previous description and the manufacturer’s method using HSP90 recombinant protein (1 μg/μL/reaction) and different concentrations of Apl-1 as ligands [21]. The thermal shift buffer (5 μL) was added, 8X Sypro Orange fluorescent dye (2.5 μL) was added, and the final volume of 20 μL was attained with distilled H_2_O. Multidrop Combi Reagent Dispenser (Thermo Fisher Scientific, Waltham, MA, USA) was used to spot the tested compounds at different concentrations on MicroAmp Optical Adhesive Film (Applied Biosystems, Foster City, CA, USA). The reactants were sealed and mixed. A Multidrop Combi Reagent Dispenser (Thermo Fisher Scientific, Waltham, MA, USA) was used to detect different concentrations of Apl-1 samples on MicroAmp Optical Adhesive Film (Applied Biosystems, Foster City, CA, USA), and the reactants were sealed and mixed. The plate temperature was heated from 25 to 99 °C at a heating rate of 1 °C/min. The detection was undertaken in the Step One Plus Real-Time PCR instrument (Applied Biosystems, Foster City, CA, USA). Displacement software (version 1.3, Applied Biosystems, Foster City, CA, USA) was used to calculate the melting temperatures (Tm) and to establish the thermal curve. The determination of Apl-1 was carried out at different concentrations in triplicate.

### 2.9. Determination of Topoisomerase II Inhibitors

The assay was performed following the manufacturer’s protocol, using a standard relaxation reaction mix (20 μL) containing 50 mM Tris-HCl (pH 8.0), 10 mM MgCl, 200 mM potassium glutamate, 10 mM dithiothreitol, 50 μg/mL bovine serum albumin, 1 mM ATP, 0.3 μg pHOT1 plasmid DNA, 8 units of human topo II (Topogen, Columbus, OH, USA), and etoposide (10 mM) or various concentrations of Apl-1, at 37 °C for 30 min. The reaction was terminated by adding 2 μL of 10% SDS, followed by 2.5 μL proteinase K (50 μg/mL) to digest the bound protein. The system was incubated at 37 °C for 15 min. The DNA product was finally analyzed by electrophoresis on a vertical 2% agarose gel at 2 volts/cm in 0.5× TAE buffer, and photographed using the Eagle Eye II system (Stratagene, La Jolla, CA, USA).

### 2.10. Statistical Analysis

All statistics are expressed as the mean ± standard deviation (SD). The comparison of statistically significant data was performed by independent Student’s *t*-test at *p* < 0.05, *p* < 0.01, and *p* < 0.001 for each experiment.

## 3. Results

### 3.1. Effect of Apl-1 on Cellular Proliferation and Migration in Cancer Cells

Previous studies on Apl-1 (Figure 1a) showed its potent cytotoxic activity against HeLa (cervical cancer cells), HCT-116 (colorectal cancer cells), NOMO-1 (acute myeloid leukemia), and HL-60 (promyelocytic leukemia cells) [40,46,47]. In this work, we used the MTT assay to evaluate the cytotoxic effect of Apl-1 on different cancer cells, including leukemia (Molt 4 and K562) and prostate cancer (PC-3 and Du145) cells, as well as on normal human skin cells (CCD966SK) and normal rat macrophage cells (NR8383). The results showed that Apl-1 exhibited potent cytotoxicity against leukemia cancer cells (Molt 4 and K562 cells with IC_50_ of 0.12 ± 0.002 and 0.54 ± 0.085 μM) and androgen-independent prostate cancer cells (PC-3 and Du145 cells with IC_50_ of 0.58 ± 0.109 and 0.33 ± 0.042 μM). For the normal rat macrophage cells NR8383 and normal human skin cells CCD966SK, Apl-1 resulted in IC_50_ of 1.54 ± 0.138 and 6.77 ± 0.190 μM, respectively, showing a weak cytotoxic activity (Figure 1b). The results indicated that Apl-1 selectively targeted cancer cells and exhibited weak cytotoxic activity against normal cells. Apl-1 showed a dose-dependent inhibitory effect on leukemia and prostate cancer cells. Apl-1 significantly inhibited the growth of the cells after 24 h at high concentrations (0.4 and 3.2 μM). The cellular inhibitory effects of Apl-1 on Molt4 and PC-3 cells were 89.6% and 75.7%, respectively (Figure 1c). The activity of Apl-1 in inhibiting prostate cancer and colony formation was tested in an in vitro cell survival test [48]. After treatment with Apl-1 (0.2 μM) for 6 h, the cells were cultured for 14 days, and the colony-formation effect of PC-3 cells was significantly reduced. In the case of Du145 cells, the colony formation was significantly reduced at a concentration of 0.4 μM (Figure 1d). Our results indicated that the cytotoxic effect of Apl-1 was cell-specific and dose-dependent.

**Figure 2:**
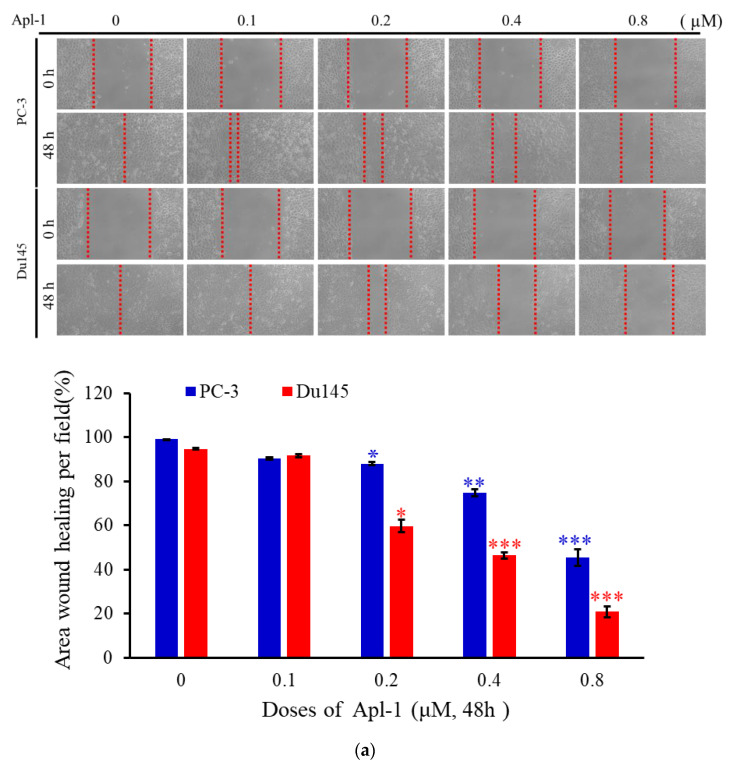

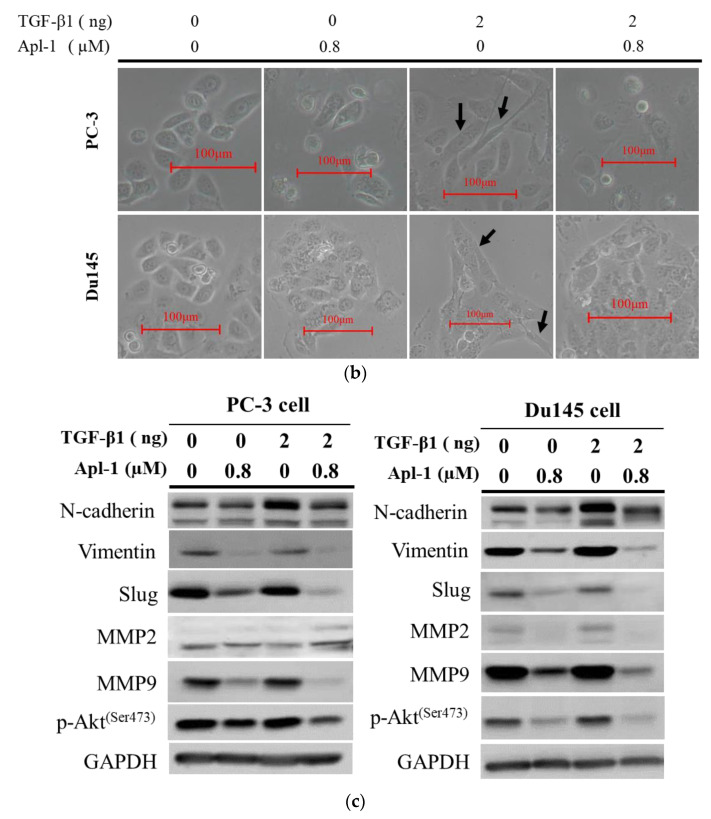
Effect of Apl-1 on the migration of PC-3 and Du145 cells and EMT activation induced with TGF-β1 in human prostate cancer. (**a**) A wound-healing assay was used and the cells were treated with different concentrations of Apl-1 for 48 h. The results were evaluated by inverted optical microscopy (100×). Each initial wound area was 100%, and the estimated wound healing was calculated as the percentage of the remaining wound area relative to each initial wound area after 48 h. The results are presented as the mean ± SD of three independent experiments. (* *p* < 0.05; ** *p* < 0.01; *** *p* < 0.001, control vs. Apl-1 group). (**b**) Apl-1 inhibited TGF- β1-induced epithelial transformation into the mesenchymal-like phenotype in PC-3 and Du145 cell lines. The cells were examined under a light microscope (200×). Arrows indicate cells with an elongated morphology. (**c**) Cells were treated with or without TGF-β1 (2 ng/mL) and co-treated with Apl-1 (0.8 µM) for 48 h, and the protein expression of EMT-associated biomarkers was determined by Western blot analysis. GAPDH acted as the loading control.

To determine the effect of Apl-1 on the migration of PC-3 and Du145 cells, the wound-healing assay was used. At concentrations of 0.2, 0.4, and 0.8 μM, Apl-1 reduced the wound closure by 6.7%, 19.8%, and 67.5% in PC-3 cells after 48 h, respectively, while in Du145 cells it reduced the wound closure by 35%, 48.4%, and 62.4%, respectively (Figure 2a). The results suggested that Apl-1 significantly inhibited PC-3 and Du145 cell migration in a concentration-dependent manner. Epithelial–mesenchymal transition (EMT) is a phenomenon by which an epithelial phenotype transforms into a mesenchymal phenotype. This phenomenon contributes to cancer metastasis [49]. Previous studies observed that Apl-1 exhibited inhibitory and regulatory effects on certain pathways, such as Wnt/β-catenin and metastatic pathway-related proteins MMP-2 [50,51]. According to a previous report, when cells were induced with TGF-β1 for EMT, the epithelial cell morphology changed from a cuboidal to an elongated spindle, and enhanced expression of the mesenchymal markers *N*-cadherin and vimentin [52]. We treated PC-3 and Du145 cells with TGF-β1 for 48 h, showing elongated cell morphology, whereas the co-treatment of Apl-1 (0.8 μM) with TGF-β1 reduced the appearance of elongated morphology in PC-3 and Du145 cells (Figure 2b). We further used a Western blot assay to detect the EMT-associated biomarkers’ protein expression. The common molecular markers of EMT are manifested by an increase in the expression of mesenchymal markers (such as *N*-cadherin and vimentin), matrix metalloproteinases (MMP-2 and MMP-9), and Slug (transcription factors) [53,54]. We used Western blot assays to analyze the protein expression of EMT-related biomarkers. Compared with the control cells, TGF-β1 treatment significantly increased the mesenchymal marker *N*-cadherin. Interestingly, the expression of vimentin protein was increased in Du145 cells under TGF-β1 treatment, but not in PC-3 cells. However, the expression of vimentin, Slug, MMP-2, and MMP-9 was significantly inhibited after co-treatment with TGF-β1 and Apl-1 (0.8 μM) (Figure 2c and Figure S1). These results indicate that Apl-1 can effectively reverse the expression of EMT biomarker proteins in PC-3 and Du145 cells induced by TGF-β1.

The molecular interaction of the PI3K/Akt signaling pathway plays an important role in the initiation of EMT. To study the potential molecular mechanism of Apl-1 inhibiting EMT in PC-3 and Du145 cells, the PI3K/Akt pathway activity was analyzed by Western blot. Treatment with 0.8 μM Apl-1 downregulated p-Akt expression in the presence or absence of 2 ng/mL TGF-β1. (Figure 2c). Apl-1 inhibited EMT through the PI3K/AKT pathway and effectively reversed the EMT induced by TGF-β1 in PC-3 and Du145 cells.

### 3.2. Apl-1 Induced Apoptosis and Interfered with Mitochondrial Membrane Potential in Leukemia and Prostate Cancer Cells

**Figure 3:**
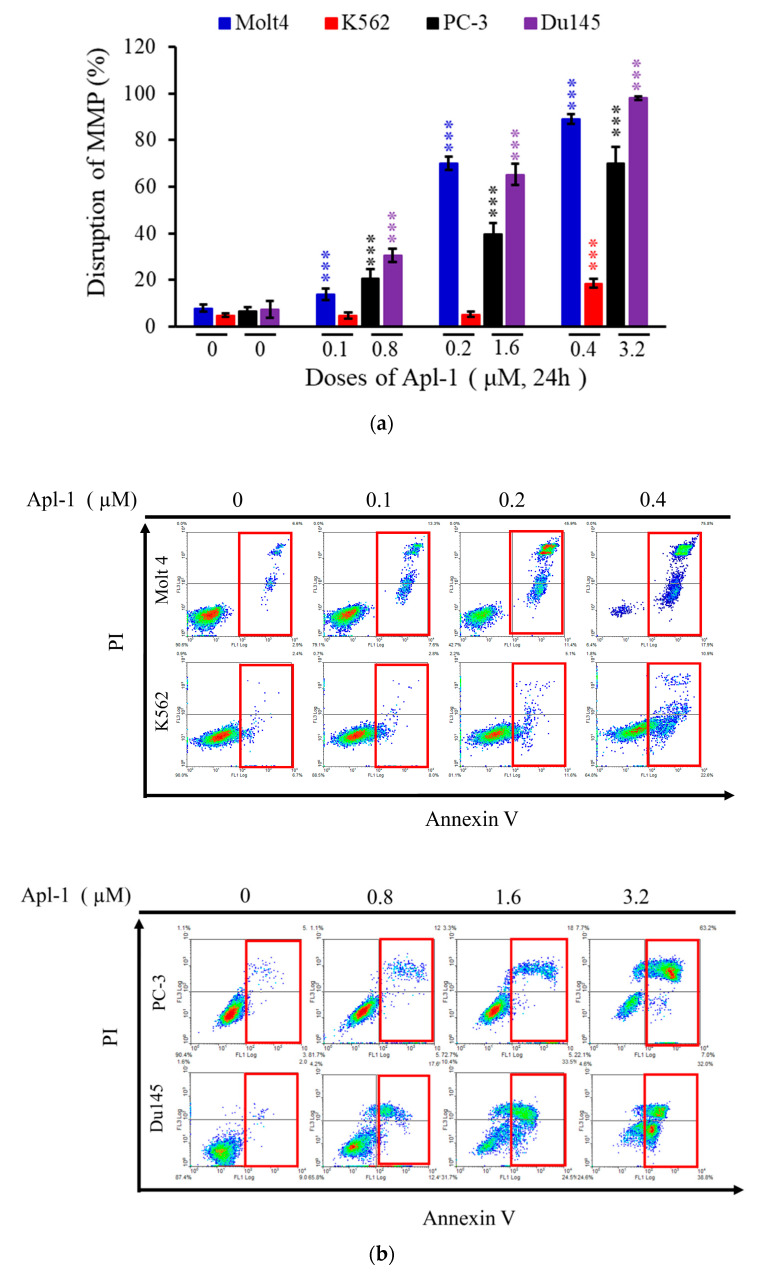

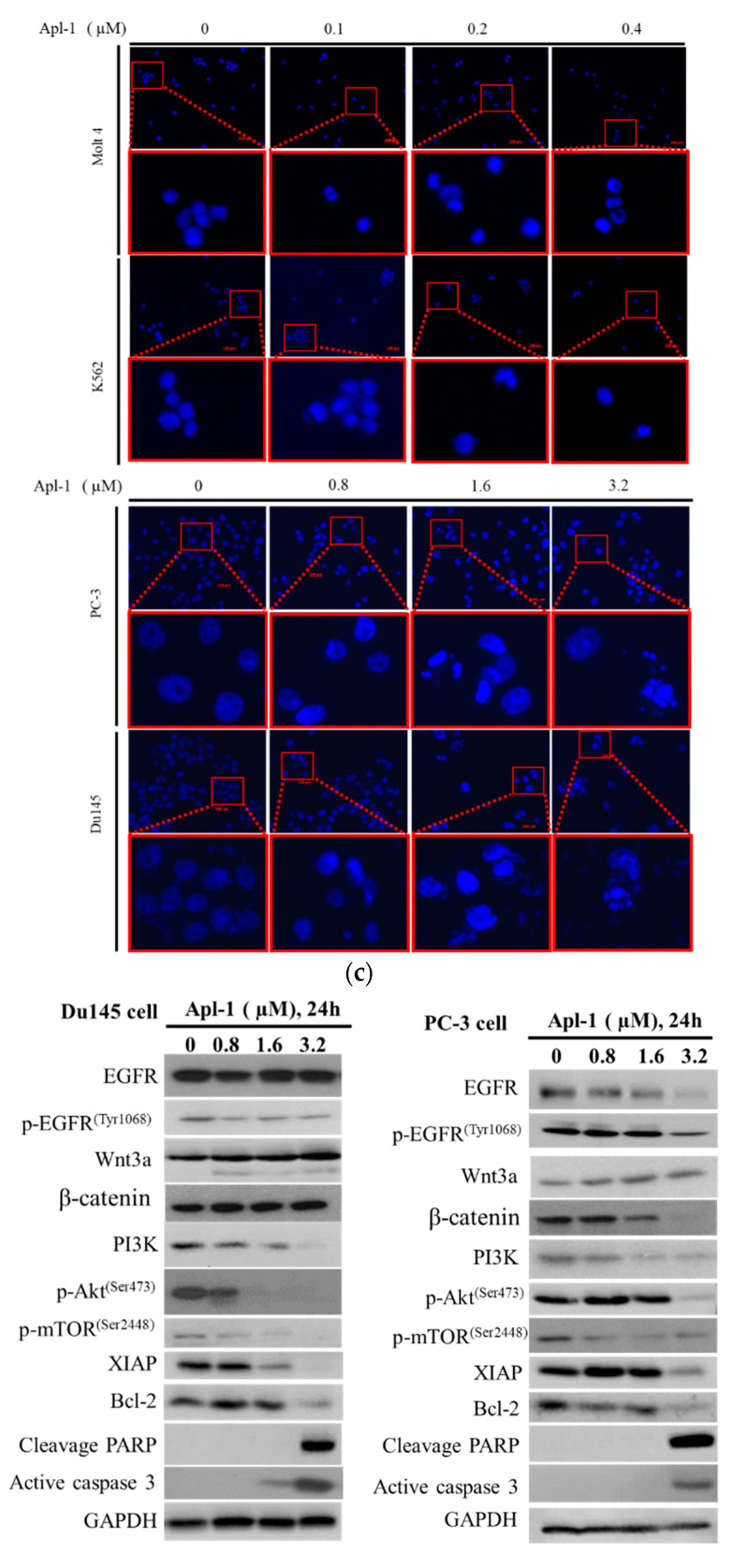

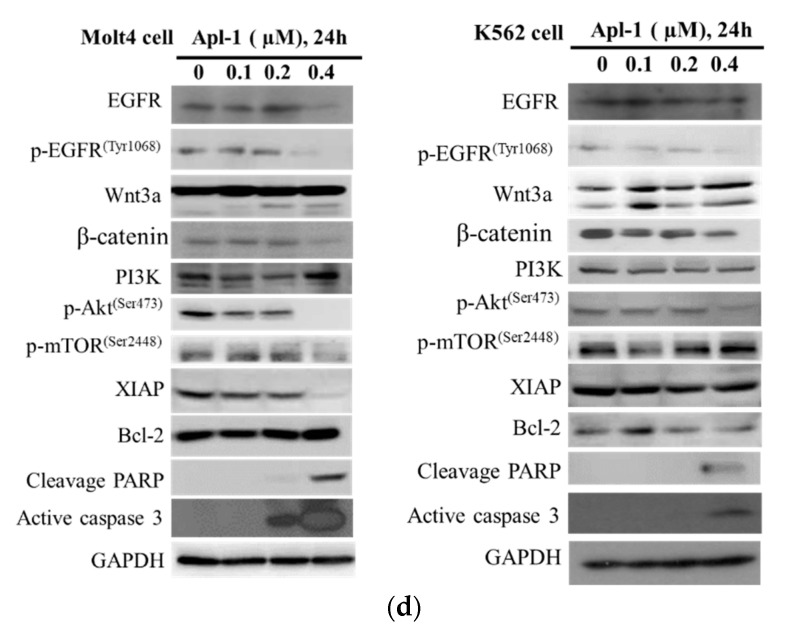
The apoptotic effect of Apl-1 on leukemia and prostate cancer cells is mediated through mitochondrial dysfunction and PI3K/AKT pathway. Cells were treated with different concentrations of Apl-1 for 24 h. (**a**) Flow cytometry analysis with rhodamine 123 staining to assess MMP destruction. (**b**) Annexin V/PI staining was used to evaluate the effect of cell apoptosis by flow cytometry analysis, and (**c**) fluorescence microscope was used to determine the changes in the nucleus morphology by DAPI staining. (**d**) The effect of Apl-1 on the expression of apoptosis-related and PI3K/AKT pathway proteins was determined with the Western blotting analysis. GAPDH acted as the loading control. The results are presented as the mean ± SD of three independent experiments. (*** *p* < 0.001, control vs. Apl-1 group).

In previous studies, Apl-1 activated caspases −8 and −9 and induced mitochondria-mediated apoptosis in endothelial cells [55]. The intrinsic pathway of apoptosis is also called the mitochondrial pathway, which is mediated by the changes in the permeability of the outer mitochondrial membrane, impaired membrane potential, assembly, and activation of the pro-apoptotic protein Bax and anti-apoptotic protein B-Cell lymphoma 2 (Bcl-2) [56]. To evaluate the effect of Apl-1 on mitochondrial membrane potential, we used flow cytometry dye rhodamine 123 stainings. The results showed that after 24 h of Apl-1 treatment, leukemia and prostate cancer cells showed a dose-dependent increase in the disruption of mitochondrial membrane potential. Molt 4, K562, PC-3, and Du145 cancer cells treated with a high Apl-1 dose (0.4 or 3.2 μM) showed a significant increase in the mitochondrial membrane potential by 89.1%, 18.6%, 71.0%, and 98.1%, respectively (Figure 3a). Then we assessed whether Apl-1 induced apoptosis in leukemia and prostate cancer cells, using annexin V/PI double staining with flow cytometry. The treatment of the four cancer cell lines with Apl-1 showed a significant dose-dependent apoptotic-inducing effect. Among the tested cell lines, the highest proportion of apoptotic cells was shown in Molt 4 cells reaching 90.8% at 0.2 μM, while the proportion of apoptotic PC-3 and Du145 cells increased by 69.6% and 70%, respectively, after 24 h of treatment with 3.2 μM of Apl-1 (Figure 3b and Figure S2a). Nucleus fragmentation and chromatin condensation are typical features of apoptotic cells. The DAPI staining with a fluorescent microscope was used to evaluate the nuclear morphological changes. In Molt4, K562, PC-3, and Du145 cells, the number of condensed nuclei and nuclear fragments was higher in cells treated with high concentrations of Apl-1 (0.4 and 3.2 μM) than in the control showing intact and normal nuclei (Figure 3c). According to previous reports, Apl-1 regulates the EGFR and PI3K pathways that play an important role in cell proliferation, apoptosis, angiogenesis, and metastasis [39,57]. Western blot analysis showed that the treatment of K562 and Molt4 cells with Apl-1 (0.4 μM) significantly increased active caspase-3 and cleaved PARP protein expression. It also decreased the expression of anti-apoptotic proteins, including p-Akt ^(ser473)^ and XIAP (Figure 3d). In prostate cancer cells, Apl-1 treatment increased PARP cleavage and activated caspase-3 expression, and inhibited the expression of anti-apoptotic proteins p-Akt ^(ser473)^, p-mTOR ^(ser2448)^, XIAP, and Bcl-2 (Figure 3d and Figure S2b). The Apl-1 (high dose) was found to significantly reduce the expression of EGFR and p-EGFR^(Tyr1068)^ protein in Molt4 and PC-3 cells, but produced no change in EGFR and p-EGFR^(Tyr1068)^ protein in K562 and Du145 cells. The abnormal activation of the Wnt signaling pathway is closely related to tumor progression and involved in various types of cancer. The classical pathway is the Wnt/β-catenin pathway, in which Wnt3a and β-catenin play important roles in regulating cell proliferation, differentiation, and development [58]. However, the results of western blot showed that Apl-1 did not significantly alter the expression of Wnt3a in Molt4, K562, PC-3, and Du145 cells, but it decreased the expression of β-catenin protein. Based on the above results, it was found that Apl-1 could induce cancer cell apoptosis by regulating the activity of anti-apoptotic proteins and increasing the expression of pro-apoptotic proteins. The apoptotic effect might involve regulation of the PI3K/AKT/mTOR pathway.

### 3.3. Apl-1 Induced NOX-Mediated ROS in Prostate and Leukemia Cancer Cells

**Figure 4:**
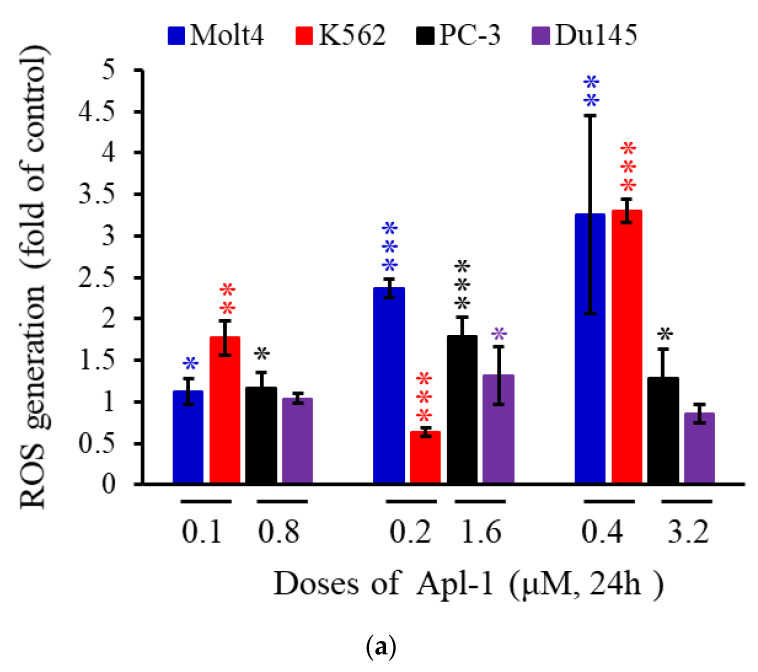

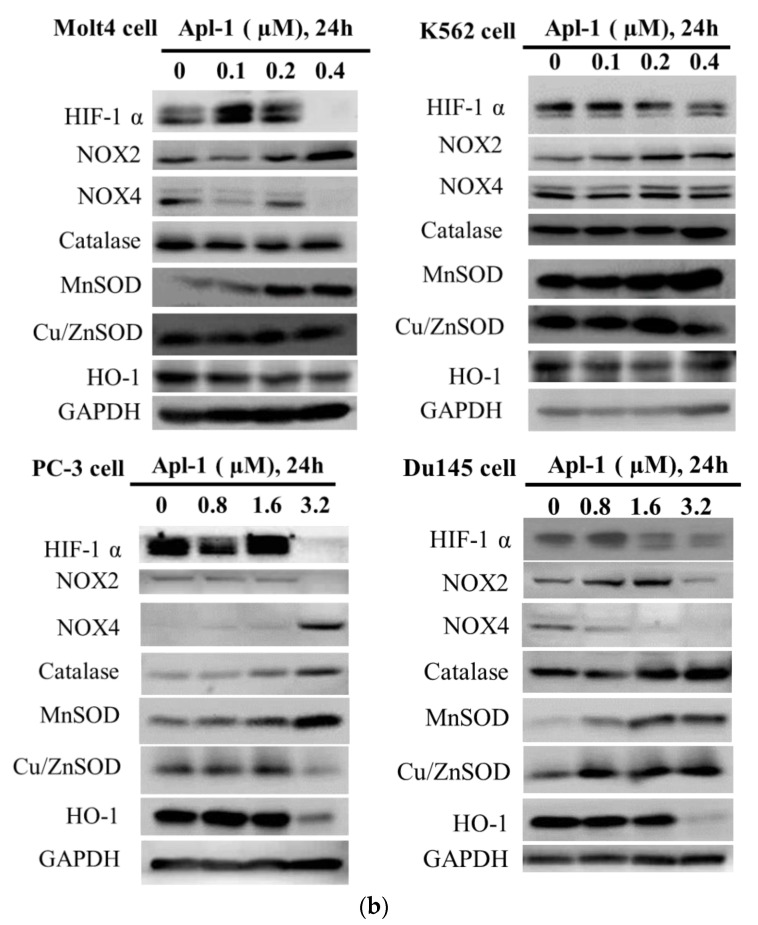
Apl-1 promoted the production of ROS by cancer cells in leukemia and prostate cancer cells. Cells were treated with various concentrations of Apl-1 for 24 h. (**a**) Flow cytometric analysis with DHE staining was used to assess ROS production. (**b**) Protein changes associated with Apl-1 intracellular oxidative regulation were evaluated by Western blot analysis. GAPDH acted as the load control. The results are presented as the mean ± SD of three independent experiments. (* p < 0.05; ** p < 0.01; *** p < 0.001, control vs. Apl-1 group).

The generation of ROS is mainly derived from complexes I and III of the mitochondrial electron transport chain or from the nicotinamide adenine dinucleotide phosphate NADPH oxidase family on the membrane, which affects the membrane permeability, resulting in mitochondria-mediated apoptosis [59,60]. Previous studies observed that Apl-1 could inhibit the peroxidase NADPH oxidase and activate superoxide dismutase (SOD) to regulate the oxidative balance of endothelial cells, but it did not affect the catalase activity [61]. To understand whether Apl-1 affects the oxidative balance of leukemia and prostate cancer cells, flow cytometry dye dihydroethidium (DHE) was used to evaluate reactive oxygen species generation in cancer cells. Molt 4, K562, PC-3, and Du145 cells were treated with a high concentration of Apl-1 (0.4 or 1.6 μM), which increased the accumulation of intracellular ROS content 4.6-fold, 3.3-fold, 1.8-fold, and 1.3-fold, respectively (Figure 4a). Our results showed that Apl-1 increased the accumulation of ROS in leukemia and prostate cancer cells. Through protein analysis, we can better understand the activity of Apl-1 in regulating the oxidative balance in cancer cells. We found that Apl-1 significantly inhibited the expression of HIF-1 α protein in prostate cancer and leukemia cells (Figure 4b and Figure S3). Apl-1 treatment reduced the expression of the antioxidant protein HO-1, and increased the expression of catalase and MnSOD in Molt 4, PC-3, and Du145 cells. Apl-1 (3.2 µM) significantly increased the expression of oxidized protein NOX4 and down-regulated the expression of NOX2 protein in PC-3 cells. Apl-1 (0.4 or 1.6 μM) upregulated the expression of NOX2 protein and inhibited the expression of NOX4 protein in Molt 4 and Du145 cells. Although Apl-1 showed different regulation mechanisms in different cells, its activity on NOX-related proteins caused cancer cells to produce too much ROS, which was accompanied by a reduction in the related antioxidant proteins.

### 3.4. The Induced Apoptosis of Apl-1 Is Mediated by Excess NOX/ROS Production

**Figure 5:**
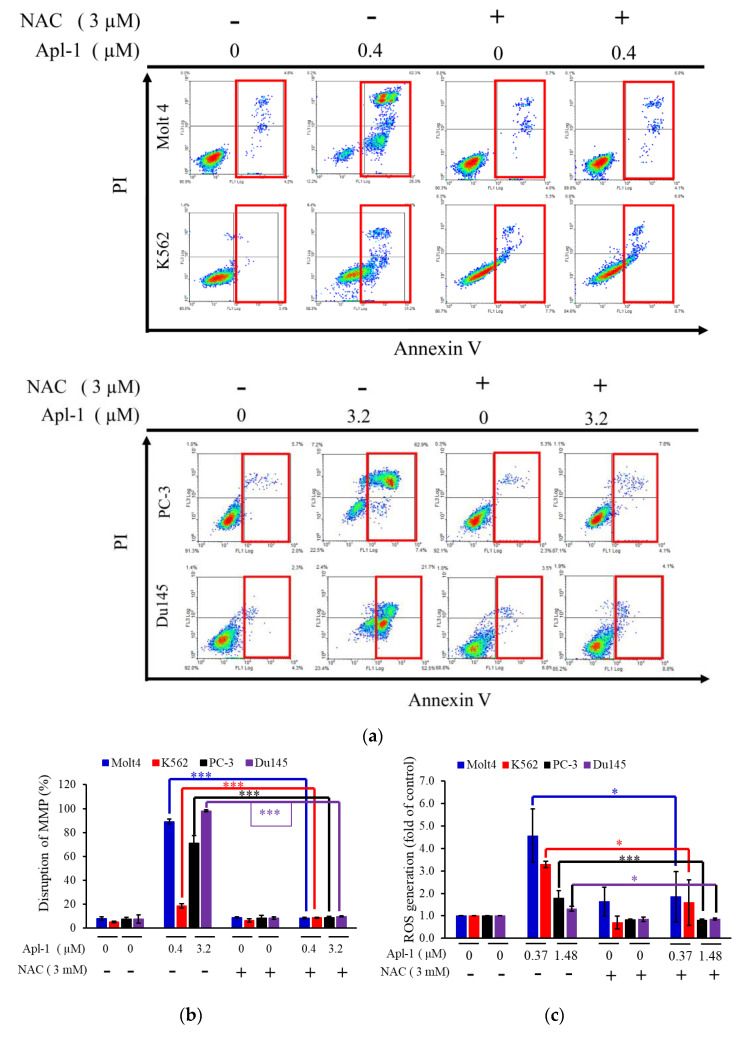

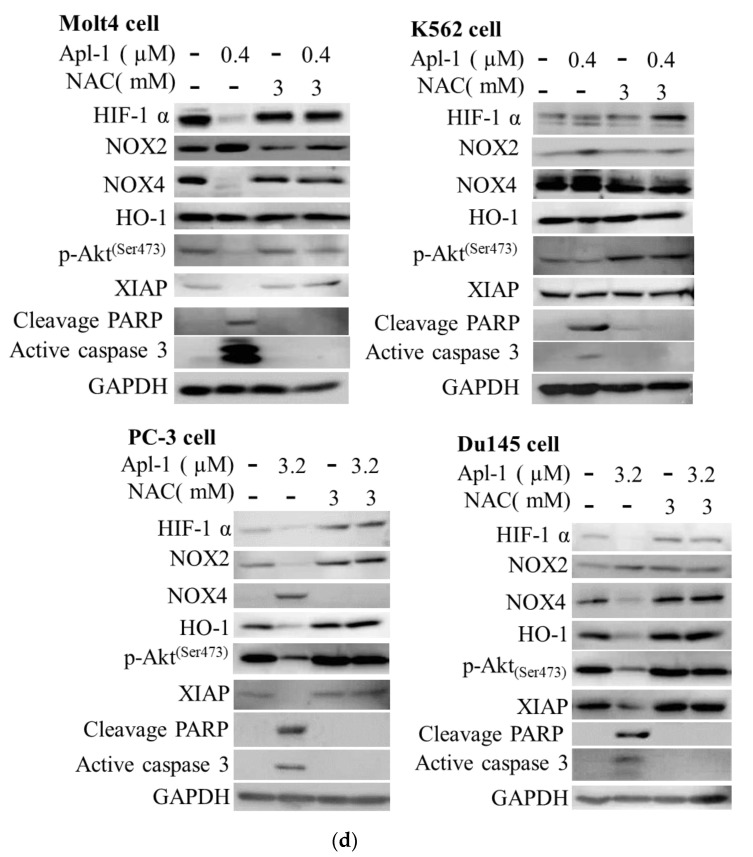
Apl-1 induced apoptosis and mitochondrial membrane potential disruption via Nox/ROS. (**a**) Leukemia and prostate cancer cells were pretreated with NAC for 30 min followed by Apl-1 for 24 h. The apoptotic population was examined with annexin-V/PI staining; the effect of the apoptotic induction with Apl-1 in cancer cells involved ROS production. The cells were treated with different doses of Apl-1 for 24 h. Leukemia and prostate cancer cells were pretreated with NAC for 30 min, followed by different doses of Apl-1 for 24 h. (**b**) The disruption of MMP and (**c**) ROS generation were examined with rhodamine 123 and DHE staining, respectively, using flow cytometry. (**d**) The effect of NAC pretreatment on the cytotoxic activity of Apl-1 was detected by Western blot assay. GAPDH acted as the load control. The results are presented as the mean ± SD of three independent experiments. (* *p* < 0.05; *** *p* < 0.001, control vs. Apl-1 group).

To confirm whether the apoptosis and mitochondrial membrane potential induced by Apl-1 are related to the accumulation of ROS in the cells, the ROS scavenger *N*-acetyl-l-cysteine (NAC) was used in the pretreatment of cells. As shown in Figure 5a and Figure S4, pretreatment with NAC significantly reduced apoptosis induced by Apl-1 by 80.7% (Molt 4), 22.1% (K562), 61.45% (PC-3), and 59.15% (Du145). The percentages of impaired mitochondrial membrane potential were, respectively, 89.1% to 8.3% and 18.6% to 8.6% in Molt 4 and K562 cells treated with Apl-1 0.4 μM after NAC pretreatment (Figure 5b). As shown in Figure 5c, pre-treatment with NAC reduced the accumulation of intracellular ROS caused by Apl-1. The above results showed that after 24 h of the pre-treatment of NAC, there was a recovery effect on both the damaged mitochondrial membrane potential and the proportion of apoptosis. The NAC pretreatment showed similar protein expression results to the negative control group. NAC pretreatment attenuated Apl-1-induced inhibition of HIF-1α and HO-1 (antioxidant-associated protein), XIAP (anti-apoptotic protein), and p-Akt expression (Figure 5d). It also reduced Apl-1-induced expression of pro-oxidative proteins, NOX2 or NOX4, and the pro-apoptotic protein cleavage of PARP and caspase-3 activation (Figure 5d). These results suggested that NAC inhibited Apl-1-induced ROS regulation of the related apoptotic pathways as well as the cellular oxidative dysregulation.

### 3.5. Interaction of Apl-1 with Topo II and Hsp90 Proteins

**Figure 6:**
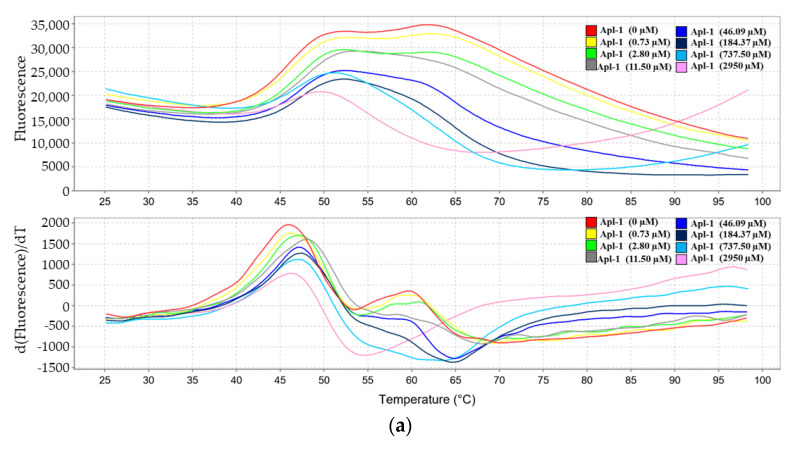

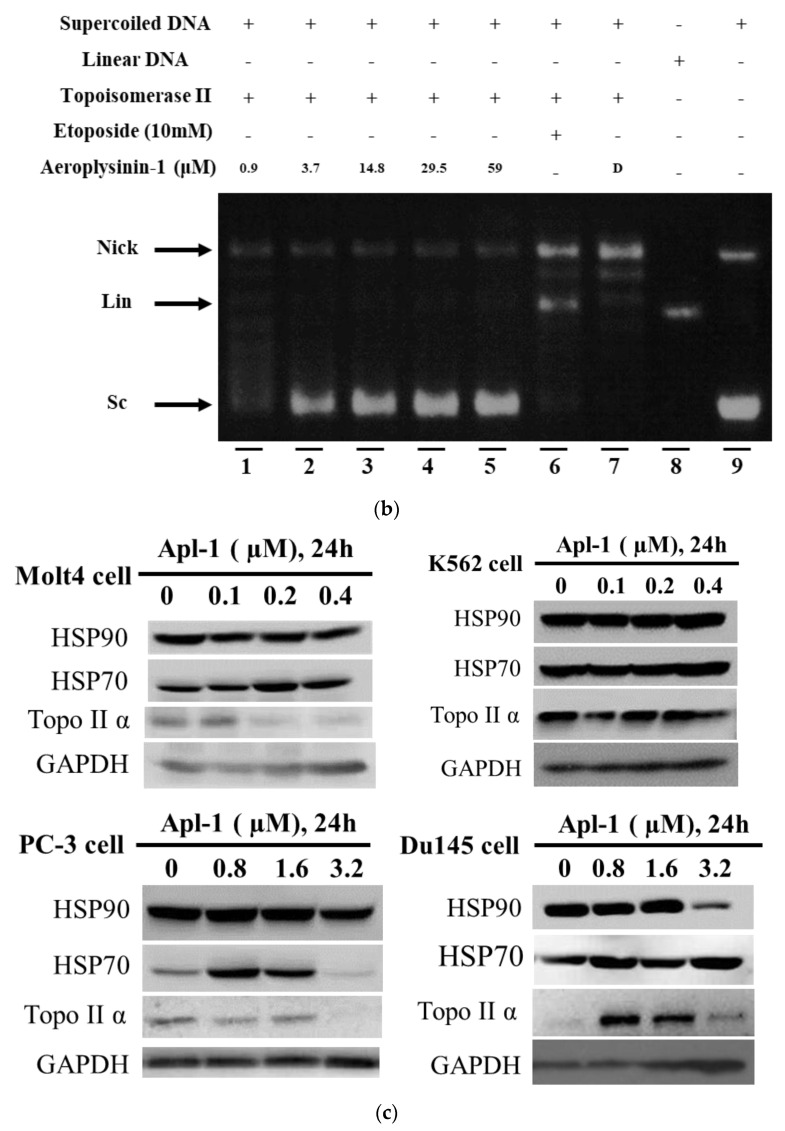
The effect of Apl-1 on the binding ability of Hsp90 and topo IIα protein. (**a**) Hsp-90 (1 μg) protein was used with different doses of Apl-1 to analyze the difference in melting temperature through fluorescence emission as a function of temperature (dF/dT). (**b**) Effects of Apl-1 on Topo II-mediated relaxation of supercoiled pHOT1 plasmid DNA in a cell-free system. Lane 6: Positive control, etoposide (10 mM), as Topo II poison (induction of linear DNA); Lane 7: Plasmid DNA + topo IIα + solvent control (induction of DNA relaxation); Lane 8: linear DNA; Lane 9: Negative control plasmid DNA (supercoiled DNA). Lanes 1–5: Apl-1 (0.9, 3.7, 14.8, 29.5, and 59 μM). Nick, nicked DNA; Sc, supercoiled DNA; Lin, linear DNA. (**c**) Apl-1 was used at different doses for 24 h, and the expression of Hsp90 and topo IIα related proteins was analyzed by Western blot.

The client proteins of Hsp90 are related to a wide range of physiological processes, including pathways such as EGFR, PI3K/AKT, and HIF-1 [62]. We evaluated the effect of Apl-1 treatment on Hsp90 and related client proteins. The protein thermal shift test was used to analyze the binding ability of Apl-1 and HSP-90 proteins. Protein thermal shift assay, also known as the different scanning fluorescence method or ThermoFluor, has been widely used to identify the influence of compounds on the stability of specific target proteins [63]. We analyzed the binding ability by gradually heating the sample (25–29 °C) and observing the changes in the fluorescence intensity. As shown in Figure 6a, the dissolution temperature of Hsp90 protein was 46.28 ± 1.49 °C, but the dissolution temperature became 92.05 ± 8.08 °C after co-treatment with Apl-1 (2950 µM). The melting temperature (Tm) of Hsp90 protein after Apl-1 treatment increased by 46.4 ± 7.4 °C. This effect could be attributed to the binding of Apl-1 with Hsp90 protein. It was previously reported that Apl-1 caused DNA damage; therefore, we used a cell-free system to evaluate the effect of Apl-1 on Topo II activity, observing DNA damage. As shown in Figure 6b, a low dose (0.9 μM) of Apl-1 induced DNA relaxation in the presence of Topo II (lane 1), but at moderate and high doses (3.7–59 μM) it inhibited the Topo II effect, converting the supercoiled DNA to the relaxed form (lanes 2–5) with an IC_50_ of 1.37 μM. Linear DNA strands were observed on supercoiled pHOT1 plasmid DNA treated with etoposide (10 mM), a standard Topo II poison (lane 6). We further used protein analysis to evaluate the Hsp90 and Topo II protein changes of Apl-1 on leukemia cells and prostate cancer cells. In leukemia cells, Apl-1 did not cause any significant changes in the expression of Hsp90 proteins, while in prostate cancer cells, at a concentration of 3.2 μM it inhibited the expression of Hsp90 proteins (Figure 6c and Figure S5). The use of 0.8 or 1.6 μM of Apl-1 in PC-3 cells significantly increased the expression of Hsp70, and the same effect also occurred in Molt 4 cells using 0.2 μM of Apl-1. The protein expression of topo IIα was inhibited in a dose-dependent manner in PC-3 and Molt4 cells. In Du145 cells, the protein expression decreased with increasing doses of Apl-1. Although Apl-1 inhibited topo IIα and Hsp90 in cell-free systems, it showed different effects in different cancer cell lines. Our results indicated that Apl-1 regulated topo IIα and Hsp90 proteins.

## 4. Discussion

Several studies have demonstrated that EMT is involved in the progression of prostate cancer metastasis and treatment resistance, and it plays a crucial role in curing mCRPC [5,64]. Many factors can regulate the effect of EMT, including the activation of transcription factors, the expression of specific cell surface proteins, and the production of extracellular matrix (ECM) degrading enzymes [65]. These factors can lead to increased invasiveness, migration ability, the production of ECM components, and the resistance of cancer cells to death. The EMT process is regulated through a variety of signaling pathways, including non-SMAD signaling involving the PI3K/Akt/mTOR pathway and SMAD and non-SMAD signals involving the HIF-1α pathway [65,66]. Our results showed that Apl-1 significantly inhibited the expression of mesenchymal cell marker proteins *N*-cadherin and vimentin, and inhibited the Slug expression of zinc-finger binding transcription factors. It also inhibited and regulated the expression of EMT-related proteins, including p-Akt and HIF-1α. (Figure 2c and Figure 4b). In TGF-β-induced EMT, Apl-1 significantly inhibited the activities of *N*-cadherin, vimentin, Slug, and p-Akt, but did not reduce the effect of HIF-1α (data not shown). Therefore, Apl-1 inhibited EMT by downregulating the activity of the PI3K/Akt/mTOR pathway.

Interestingly, previous reports showed that Apl-1 (20 μM) reduced the ROS content in RF-24 endothelial cells [61]. In this study, it was found that Apl-1 increased the accumulation of ROS in prostate cancer and leukemia cells (Figure 4a). There are several stains to detect intracellular ROS, and different types of ROS can cause different results. The previous literature used 2,7-dicholorodihydrofluorescein diacetate (DCFH-DA) to detect the intracellular H_2_O_2_ content, but in this work, DHE was used to evaluate superoxide production [67]. This led to different results reporting the accumulation of ROS in cells: Apl-1 may cause superoxide accumulation in cells, but not H_2_O_2_ superoxide accumulation. The treatment of RF-24 endothelial cells with Apl-1 inhibited NADPH oxidase activity and significantly increased SOD activity, but did not show any relevant effect on catalase [61]. Our results indicated that Apl-1 treatment activated the protein expression of NOX4 and NOX2 in prostate cancer cells PC-3 and Du145, respectively (Figure 4b). Prostate cancer cells treated with Apl-1 showed a significant increase in the expression of mitochondrial-related MnSOD proteins, while it showed significant inhibition of the expression of antioxidant protein HO-1. Therefore, the accumulation of ROS in prostate cancer cells by Apl-1 might be attributed to the increase in the expression of the pro-oxidant protein NOX and the decreased expression of the antioxidant protein HO-1.

Several previous reports demonstrated that Apl-1 induced apoptosis in cancer cells by multiple mechanisms. The results of our cell-free system and Western blot analysis indicated that Apl-1 can regulate topo II and Hsp90 activity (Figure 6). Although many drugs targeting topo II achieved clinical success, the development of drug-resistant cancer cells may limit their clinical efficacy [22]. Therefore, it is necessary to develop dual inhibitors with cancer-related targets, and Apl-1 has the potential to be developed as a dual inhibitor of topo II and Hsp90. Furthermore, the oxidized derivative of aeroplysinin-1 (1′R,5′S,6′S)-2-(3′,5′-dibromo-1′,6′-dihydroxy-4′-oxocyclohex-2’-enyl) acetonitrile (DT) acted as a catalytic inhibitor of topoisomerase II [38]. To better understand the effect of aeroplysinin-1 and its related derivatives on topoisomerase II activity, in future research, we will use a cell-free system assay to examine the effect of aeroplysinin-1 and its related derivatives on topoisomerase II activity. The structure of the compounds and the active site relationship of topoisomerase II will be studied by molecular docking simulation.

ROS possess different functions at different stages in cancer progression and are involved in the regulation of multiple cell survival pathways, including PI3K/AKT/mTOR and MAPK/ERK mitotic signaling to drive cell proliferation [25]. In this study, Apl-1 caused a significant impairment in mitochondrial membrane potential and increased intracellular ROS generation. The pretreatment with NAC effectively prevented the impaired mitochondrial membrane potential and intracellular ROS generation (Figure 5a–c). Pretreatment with NAC reduced the expression of Apl-1-activated caspase-3 and PARP cleavage and restored XIAP and p-Akt expression. It also restored the antioxidant proteins HO-1 and HIF-1 and reduced NOX expression (Figure 5d). Our results suggested that the Apl-1-induced apoptosis involved the PI3K/AKT pathway through the activation of NOX and the downregulation of HO-1 and HIF-1 proteins.

Our study showed that the isoxazoline bromide alkaloid, Apl-1, was expressed in human prostate cancer and leukemia cells by inducing ROS-mediated mitochondria-dependent apoptosis. Apl-1 induced ROS production from different sources, including NOX activity, mitochondrial release, and inhibition of HIF-1α and HO-1 antioxidant-related proteins. Pretreatment with the ROS scavenger, NAC, simultaneously attenuated the apoptosis and mitochondrial membrane potential disruption of Apl-1, and restored the activities of p-Akt, HIF-1α, and HO-1 inhibited by Apl-1. Therefore, Apl-1 was suggested to regulate NOX, HIF-1a, and HO-1, resulting in ROS generation rather than mitochondrial release. In addition, EMT plays an important role in the regulation of cancer metastasis and invasion, and also regulates the generation of cancer stem cells (CSCs) [68]. In this study, we revealed for the first time that Apl-1 inhibits EMT in prostate cancer cells and inhibits EMT induced by TFG-β. Collectively, these results suggested that Apl-1 promoted dysregulated oxidative stress in NOX and HIF-1α signaling pathways, leading to the induction of apoptosis via the downregulation of the PI3K/Akt signaling pathway.

## 5. Conclusions

The treatment of leukemia and prostate cancer cells with the isoxazoline bromide alkaloid aeroplysinin-1 (Apl-1) induced mitochondrial dysfunction and ROS generation, leading to apoptosis. In the cell-free system assay, Apl-1 showed potent inhibition of topoisomerase II activity, with an IC_50_ value of 1.37 μM. The Western blot analysis showed that Apl-1 exhibited a dose-dependent inhibition of topoisomerase II activity in Molt4 and PC-3 cells. Fluorescent protein thermal shift assays and Western blot analysis were used to identify the interaction of Apl-1 and Hsp90. These results suggested that Apl-1 has potential as a multi-target inhibitor. The results in the presence or absence of TGF-β showed that Apl-1 could inhibit EMT through the PI3K/AKT signaling pathway. Pretreatment with *N*-acetylcysteine (NAC) reduced Apl-1-induced mitochondria-dependent apoptosis and restored NOX, HO-1, and HIF-1a expression. Taken together, our results suggested that Apl-1 contributed to cellular oxidative stress by activating NOX, inhibiting HIF-1α and HO-1 dysregulation, and promoting mitochondria-dependent apoptosis. Our findings revealed that Apl-1 targeted different cancer-related signaling pathways, and could be developed as a drug lead against prostate cancer and leukemia cancer cells.

## Data Availability

Not applicable.

## Supplementary Materials

The following supporting information can be downloaded at: https://www.mdpi.com/article/10.3390/life12050687/s1, Figure S1: Quantitative western blot analysis to determine protein expression of EMT-related biomarkers.; Figure S2: The apoptotic effect of Apl-1 on leukemia and prostate cancer cells is mediated through mitochondrial dysfunction and PI3K/AKT pathway.; Figure S3: Apl-1 promoted the production of ROS by cancer cells in leukemia and prostate cancer cells.; Figure S4: Apl-1 induced apoptosis via Nox/ROS.; Figure S5: The effect of Apl-1 on the binding ability of Hsp90 and topo IIα protein.
