# Supplementary material for: The Antileukemic and Anti-Prostatic Effect of Aeroplysinin-1 Is Mediated through ROS-Induced Apoptosis via NOX Activation and Inhibition of HIF-1a Activity
Source: Life (Basel). 2022 May 5;12(5):687. doi: 10.3390/life12050687 (PMC9145196; doi:10.3390/life12050687)
Supplement: Supplementary file 1 [file life-12-00687-s001.zip › life-1668008-supplementary.pdf]

Supplementary Data

1. Supplementary Figures and Tables

1.1. Effect of Apl-1 on the migration and EMT activation-induced with TGF-β1 in human prostate cancer.

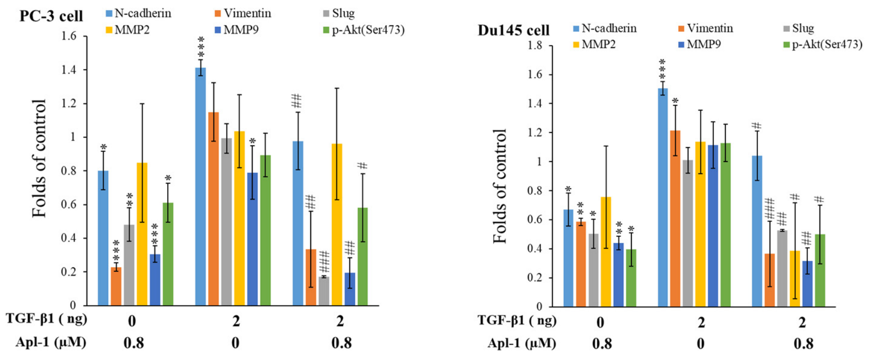

Supplementary Figure S1. Quantitative western blot analysis to determine protein expression of EMT-related biomarkers. The results are presented as the mean ± SD of three independent experiments. (**p < 0.05; **p < 0.01; ***p < 0.001, control vs Apl-1 group*). Compared with the group containing TGF-β1(*#p < 0.05; ##p < 0.01; ###p < 0.001*, TGF-β1 and Apl-1 co-treatment group).

1.2. Apoptotic effect of Apl-1 on leukemia and prostate cancer cells

(a)

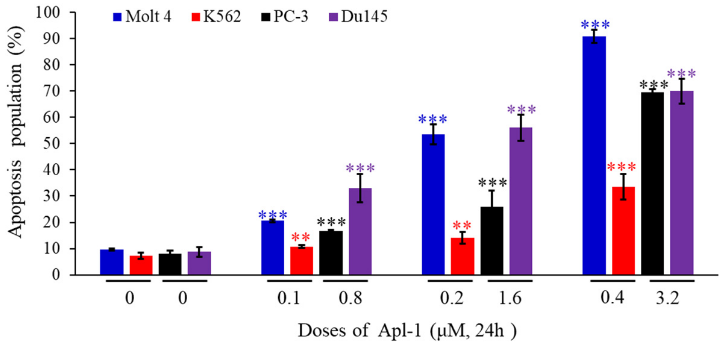

(b)

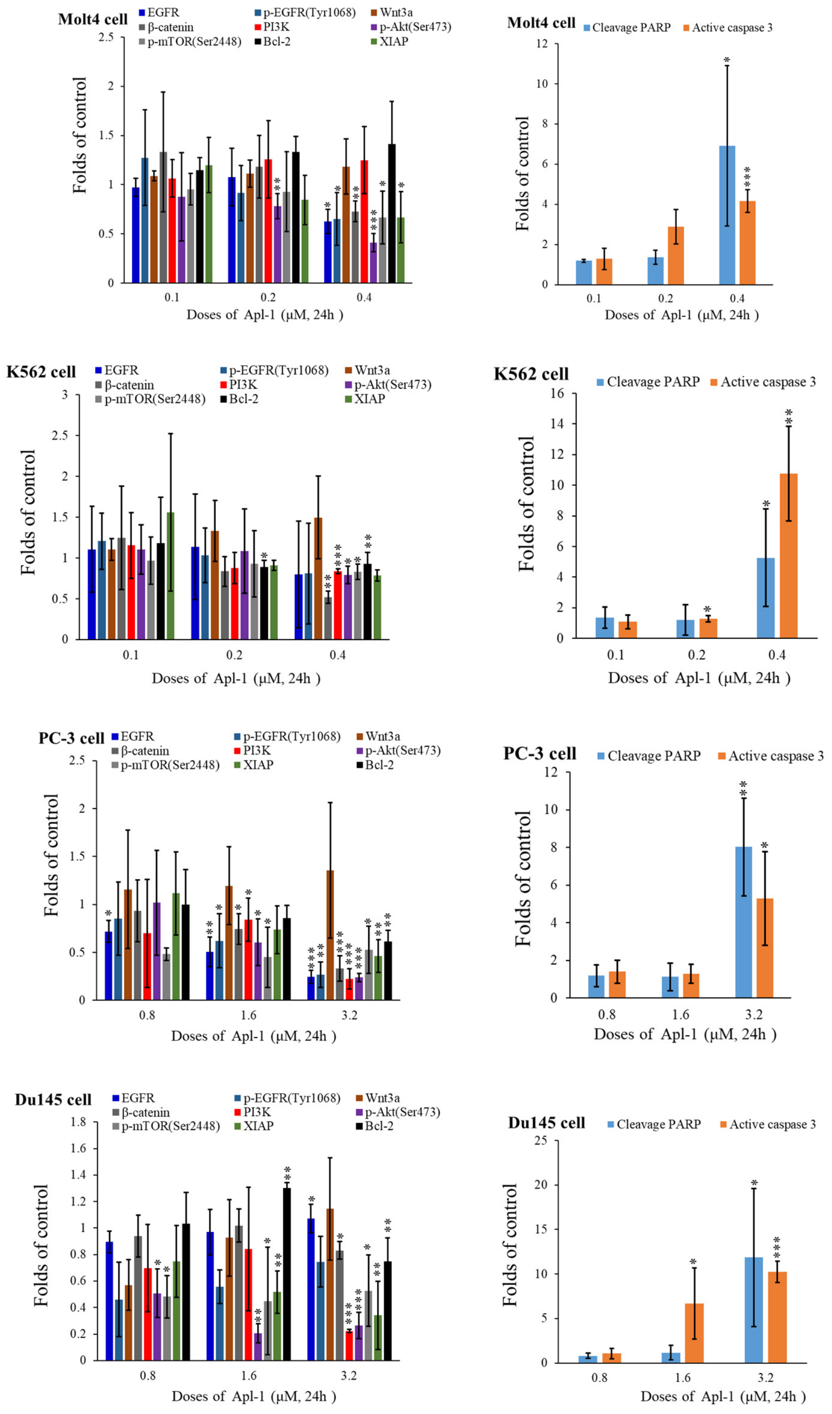

Supplementary Figure S2. The apoptotic effect of Apl-1 on leukemia and prostate cancer cells is mediated through mitochondrial dysfunction and PI3K/AKT pathway. (a)Quantify the percentage of apoptosis marked by Annexin V/PI staining. (b) Quantification of densitometric values of apoptosis-related proteins and PI3K/AKT pathway proteins. (**p* < 0.05;

$^{**}p < 0.01$; $^{***}p < 0.001$, control vs Apl-1 group).

1.3. Apl-1 induced NOX-mediated ROS in prostate and leukemia cancer cells

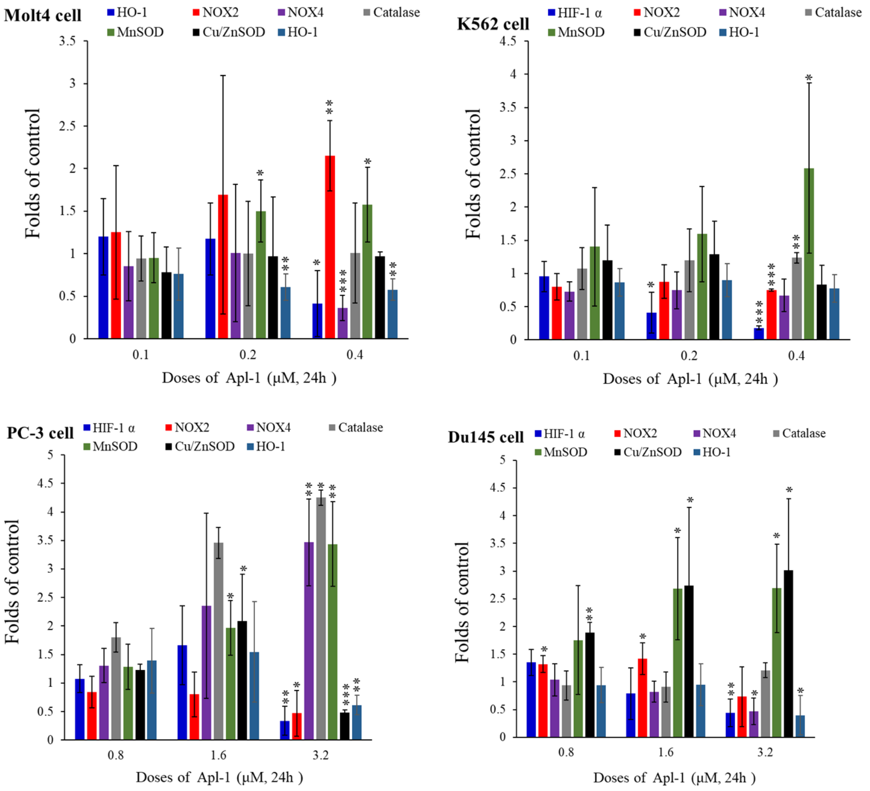

Supplementary Figure S3. Apl-1 promoted the production of ROS by cancer cells in leukemia and prostate cancer cells. Densitometric values to quantify protein changes associated with intracellular oxidative regulation. (*$^{*}p < 0.05$; $^{**}p < 0.01$; $^{***}p < 0.001$, control vs Apl-1 group*).

1.4. The induced apoptosis of Apl-1 is mediated by excess NOX/ROS production.

(a)

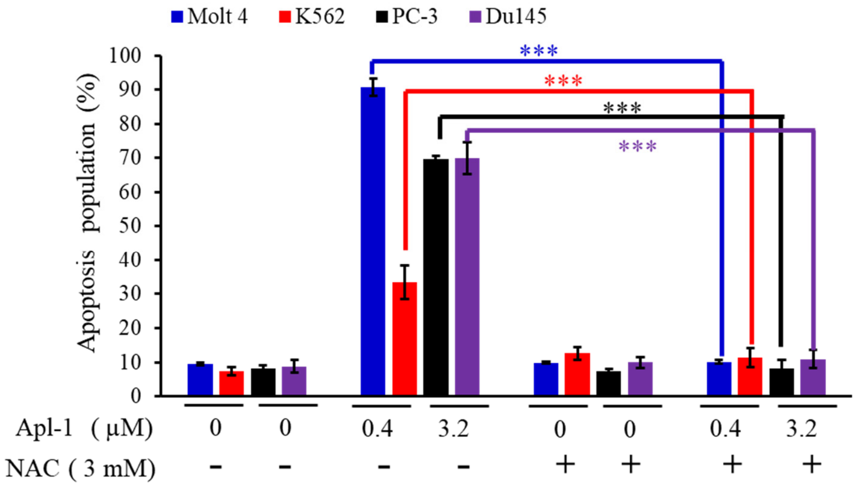

(b)

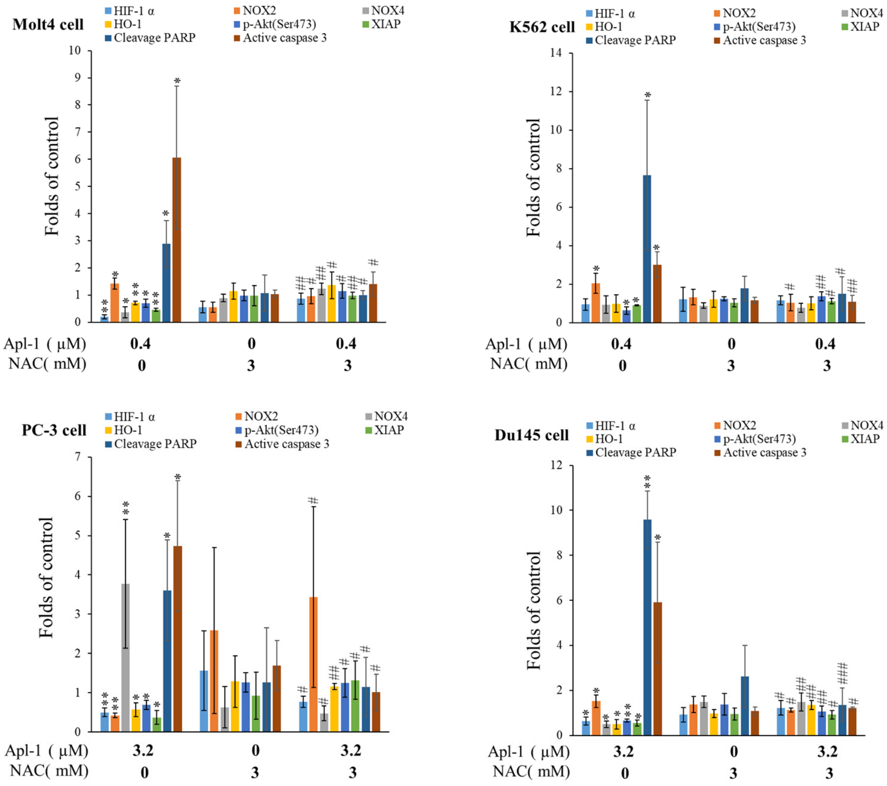

Supplementary Figure S4. Apl-1 induced apoptosis via Nox/ROS. (a) To quantify the percentage of apoptotic populations that were pretreated with NAC for 30 min and then treated with different doses of Apl-1 for 24 h.(b) Quantitative Western blot was used to detect the NOX/ROS-mediated apoptosis-related proteins regulated by NAC pretreatment on Apl-1. (*p <

*0.05; **p < 0.01; ***p < 0.001, control vs Apl-1 group*). Compared with the group containing NAC (*#p < 0.05; ##p < 0.01; ###p < 0.001*, NAC and Apl-1 co-treatment group).

1.5. Interaction of Apl-1 with Topo II and Hsp90 proteins

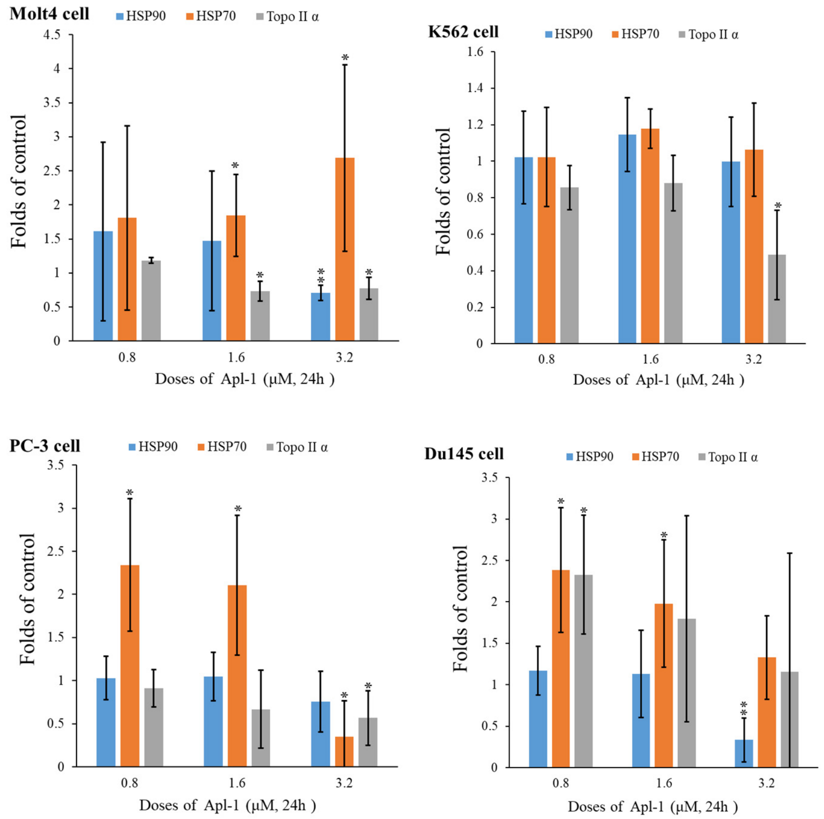

Supplementary Figure S5. The effect of Apl-1 on the binding ability of Hsp90 and topo IIα protein. Quantification of expression of Hsp90 and topo IIα-related proteins was analyzed by western blotting.
